# Meeting of the State Association of Health Officers

**Published:** 1911-12

**Authors:** 


Meeting of the State Association of Health Officers.—A meeting of the county and city health officers of Texas in session with the State Board of Health was held in Austin, Thursday, November 16 ult., at which there was a slim attendance,—only about twenty-five being present. Dr. Ralph Steiner, State Health Officer and ex-officio president of the State Board of Health and of the Association of Health Officers, presided. Other than the president there were only two members of the State Board present, Dr. Calhoun of Beaumont and Dr. Beall of Fort Worth. This is regrettable, as showing a lack of interest in the important questions to be discussed and settled. Some highly interesting papers were read, however, by Dr. Trueheart of Galveston, Dr. Burg of San Antonio, Dr. Calhoun of Beaumont and others whom I do not now recall. These papers will be published in the Bulletin of the State Board.

Apropos of hook worm, Dr. Steiner gave out for publication the following:

"Hook worm disease, known technically as uncinariasis, is a very common disease in the tropical and subtropical belts of the entire world. It is caused by small parasites, nearly half an inch long, which live in the intestinal canal. We know of two different forms of hook worms in man, one known as the old world hook worm, discovered in 1843 by Dr. Dubini of Italy, the other, known as the new world hook worm, recognized in 1902 by Dr. Stiles of the United States Public Health and Marine Hospital Service.

"Prior to 1902 this disease was known in many different parts of the world, but not until 1902 was it known to be common in the United States, when, almost simultaneously, Professor Allen J. Smith of the medical department of the University of Texas published eight cases of the infection found among the medical students at Galveston; Dr. Harris, secretary of the State Board of Health of Georgia, published the claim that it was the most common of the infectious diseases of the South, and Professor Stiles of the Federal service published the fact that our American parasite was distinct from that known in Europe, and he demonstrated the presence of this new parasite in Virginia, North Carolina, South Carolina, Georgia, Florida and Texas. It was then also that Stiles put forward the startling claim that the condition of the tenant white class (sometimes unfortunately called the poor whites) in the South was directly due to this infection. Following the claims of Smith, Harris and Stiles, the ridicule and indignation of the country turned against Stiles because of his claim that the tenant white problem was primarily a medical problem and that hook worms were at the basis of the condition of these people."

